# Effects of Chilling Stress on Morphological, Physiological, and Biochemical Attributes of Silage Corn Genotypes during Seedling Establishment

**DOI:** 10.3390/plants11091217

**Published:** 2022-04-29

**Authors:** Jiaxu Wu, Muhammad Nadeem, Lakshman Galagedara, Raymond Thomas, Mumtaz Cheema

**Affiliations:** School of Science and the Environment, Grenfell Campus, Memorial University of Newfoundland and Labrador, Corner Brook, NL A2H 5G4, Canada; lgalagedara@grenfell.mun.ca (L.G.); rthomas@grenfell.mun.ca (R.T.)

**Keywords:** antioxidant, cold stress tolerance, plant growth, photosynthesis, root morphology, silage corn

## Abstract

Chilling stress is one of the major abiotic stresses which hinder seedling emergence and growth. Herein, we investigated the effects of chilling/low temperature stress on the morphological, physiological, and biochemical attributes of two silage corn genotypes during the seedling establishment phase. The experiment was conducted in a growth chamber, and silage corn seedlings of Yukon-R and A4177G-RIB were grown at optimum temperature up to V3 stage and then subjected to five temperature regimes (25 °C as control, 20 °C, 15 °C, 10 °C, and 5 °C) for 5 days. After the temperature treatment, the morphological, physiological, and biochemical parameters were recorded. Results indicated that temperatures of 15 °C and lower significantly affected seedling growth, photosynthesis system, reactive oxygen species (ROS) accumulation, and antioxidant enzyme activities. Changes in seedlings’ growth parameters were in the order of 25 °C > 20 °C > 15 °C > 10 °C > 5 °C, irrespective of genotypes. The chlorophyll content, photosynthetic rate, and maximal photochemical efficiency of PS-II (*F_v_*/*F_m_*) were drastically decreased under chilling conditions. Moreover, chilling stress induced accumulation of hydrogen peroxide (H_2_O_2_)and malonaldehyde (MDA) contents. Increased proline content and enzymatic antioxidants, including superoxide dismutase (SOD), catalase (CAT), and ascorbate peroxide (APX), were found to alleviate oxidative damage under chilling stress. However, the genotype of Yukon-R exhibited better adaption to chilling stress than A4177G3-RIB. Yukon-R showed significantly higher proline content and enzymatic antioxidant activities than A4177G3-RIB under severe chilling conditions (temperature ≤ 10 °C). Similarly, Yukon-R expressed low temperature-induced ROS accumulation. Furthermore, the interaction effects were found between temperature treatment and genotype on the ROS accumulation, proline content and antioxidant enzyme activities. In summary, the present study indicated that Yukon-R has shown better adaptation and resilience against chilling temperature stress, and therefore could be considered a potential candidate genotype to be grown in the boreal climate.

## 1. Introduction

Silage corn (*Zea mays* L.) is known as a high-energy crop, with higher biomass production potential, uniform growth quality, and one-cut harvest benefits [1,2,3]. Moreover, silage corn is highly palatable, digestible, and easy to ensile due to the high content of soluble sugar [4]. Due to these attributes, silage corn is mostly cultivated in large acreages as a forage crop [5,6]. Corn originates from subtropical regions and is highly sensitive to chilling stress (0–20 °C) at all growth and development stages [7,8]. However, chilling stress usually occurs in the short growing season in the boreal climate where the average seasonal growth temperature ranges from 10 to 12 °C, which is a major environmental factor limiting forage yield [9,10].

Chilling stress induces significant physiological and morphological changes in growing plants, including reduced seedling emergence, leaf initiation, disordered root cell division, and elongation [11,12]. The reduction in root architectural growth also results in reduced water and nutrient uptake, leading to reduced nutrient use efficiency [13]. Moreover, chilling temperatures delay flowering and disturb the gametophyte and pollen development during reproductive growth, and such effects contribute to poor seed filling and seed development [14,15,16]. 

Numerous studies have indicated that plant photosynthetic capacity is inhibited by chilling stress, which can cause dysfunction of photosynthesis apparatus localized inside the chloroplast [17,18]. The stomatal closure has negative effects on the leaf gas exchange due to the limitation of CO_2_ supply [19,20]. The low-temperature stress also decreases light absorption in the thylakoid electron transport of photosystem II (PS-II) and increases the excitation energy quenching in the light-harvesting antennae [21]. Adam and Murthy [22] and Sharma et al. [23] demonstrated the decreased leaf maximum photochemical efficiency of PS-II (*F_v_*/*F_m_*), the efficiency of excitation energy transfer to open PS-II reaction center (*F_v_’*/*F_m_’*), and non-photochemical quenching coefficient (q_p_) values and photoinhibition of PS-II in the growing plants under chilling stress. 

Chilling stress also contributes to the over-accumulation of reactive oxygen species (ROS) in the growing plants [24]. Generally, ROS such as hydrogen peroxide (H_2_O_2_), superoxide (O_2_^•−^), singlet oxygen (^1^O_2_), and hydroxyl radical (OH•) are produced under chilling stress, and such ROS over-accumulation leads to plant oxidative stress [8,25,26]. It has been reported that lipid peroxidation increases membrane leakage, whereas it decreases the membrane fluidity and is considered as the main reason for damaging membrane-localized proteins impacting ion channels, receptors, and enzymes functions [27]. Moreover, chilling stress affects the stability and solubility of proteins which leads to disrupted metabolic reactions for plant growth and development including the Calvin cycle [28].

Plants, therefore, attempt to mediate the adverse effects of temperature downshifts through stress tolerance defense mechanisms [29]. Plants increase their chilling stress tolerance by regulating cold-induced genes expressions, osmotic potential, membrane stabilization, and ROS scavenging activation [30,31]. For instance, the C-repeat binding factor/dehydration-responsive element-binding protein 1 (CBF/DREB1) dependent signal pathway was reported to show a complex regulation of the cold response network [32]. For instance, the *CBF* genes are reported to bind to conversed CRT/DRE motifs (A/GCCGAC) in the promoter of *COR* genes and then induce their expression to enhance cold stress [33,34,35]. The biosynthesis of osmoprotectants such as soluble sugars, polyamines, glycine betaine (GB), and proline is also considered a strategy used by plants to adapt to chilling stress [36]. In addition, plants require detoxifying systems comprising enzymatic and non-enzymatic antioxidants to reduce the ROS accumulation during chilling stress conditions [37]. Enzymatic antioxidants of plants include superoxide dismutase (SOD), catalase (CAT) and ascorbate peroxide (APX), glutathione peroxidase (GPX), and others [27]. Previous studies showed that plant chilling tolerance capacity is linearly correlated with antioxidant systems, and the transcript levels of antioxidant enzymes are upregulated in chilling-tolerant varieties [27,38,39]. These results indicate the crucial role of the antioxidant system in alleviating oxidative stress during chilling temperature conditions. In addition, low-temperature stress tolerance varies within crop species and genotypes. Our previous studies showed that the agronomic performance varies in silage corn genotypes when evaluated at the physiological maturity under field conditions in the boreal climate [9,40,41]. Early seedling establishment is an important step in crop growth and cold/chilling stress is very common in boreal climates. There is limited literature available on plant growth, photosynthetic characteristics, and antioxidant activities in silage corn genotypes under low-temperature/chilling stress during the early growing stages, particularly in boreal agro-ecosystems. Therefore, the aim of this study was to assess the effects of low-temperature regimes on morphological, physiological, and biochemical responses of two contrasting silage corn genotypes. Furthermore, we tested the relationships between the growth parameters and chilling stress tolerance parameters of silage corn genotypes. We hypothesized that low-temperature/chilling stress would alter root morphological traits, shoot growth, photosynthetic efficiency, redox homeostasis, and enzymatic antioxidants of silage corn. Moreover, the expression of these traits in silage corn genotypes would contribute to enhancing chilling tolerance differentially at the early growth stage.

## 2. Materials and Methods

### 2.1. Experimental Design, Plant Material, and Growth Conditions 

The experiment was conducted in a walk-in growth chamber (BioChambers Inc., Winnipeg, MB, Canada) at Grenfell Campus, Memorial University of Newfoundland and Labrador, Canada in 2020/2021. The experiment was set up in a completely randomized design in a split-plot arrangement with three replications and repeated twice. The experimental treatments included two silage corn genotypes (Yukon-R and A4177G3-RIB) and five temperature levels (25 °C as control, 20 °C (non-chilling), and 15 °C, 10 °C, and 5 °C were considered as chilling temperatures). 

Silage corn genotypes were selected based on our previous published work where we demonstrated that Yukon-R performed better than A4177G3RIB in terms of agronomic performance [9,10,40,41]. Equal sized silage corn seeds with homogeneous seed weight (0.24~0.28 g) were selected for the experiment. The sterilized silage corn seeds were then placed on a plastic tray with water-saturated tissue paper in the dark at 25 °C for germination [42]. The seeds were allowed to germinate for 4 days, and water was sprayed daily to keep the tissue paper at a saturated moisture level. After the germination, the seedlings with uniform size were anchored in a styrofoam board and transferred to plastic containers (60 cm × 40 cm × 30 cm) containing half-strength Hoagland’s nutrient solution [43]. The pH of the nutrient solution was maintained between 5.8 and 6.2 and the nutrient solution was changed every fourth day. The growth chamber settings were tuned to 25 °C, with 14 h/10 h (light/dark) photoperiod, 65–70% relative humidity, and 300 μmol m^−2^ s^−1^ light intensity. The growth condition of silage corn seedlings was monitored daily, and temperature and humidity conditions of the growth chamber were recorded by the HOBO data logger (Onset Computer Corporation, Bourne, MA, USA). At the V3 stage (10-day old seedling), the temperature regime was imposed according to the treatments (optimum or chilling stress). There were three containers for each temperature treatment, each container represents one replication with 20 seedlings (10 seedlings for each genotype). 

### 2.2. Evaluation of Plant Growth Performance

#### 2.2.1. Photosynthesis Rate and Maximum Photochemical Efficiency of Photosystem-II

Photosynthesis rate was measured from the second fully expanded seedling leaf from the top using a portable LI-COR 6400XT photosynthesis system (LI-COR BioSciences, Lincoin, NE, USA) maintaining the leaf chamber temperature, air relative humidity, CO_2_ concentration, and photosynthetic photon flux density (PPFD) at 20 °C, 80–90%, 400 μmol mol^−1^, and 1000 μmol m^−2^ s^−1^, respectively. The maximum photosystem II (PS-II) quantum yield (*F_v_*/*F_m_* = (*F_m_* − *F*_0_)/*F_m_*) of the seedling leaves was recorded using the LI-6400XT with leaf chamber fluorometer after 30 min of dark adaption [44]. 

#### 2.2.2. Plant Growth and Root Characteristics

Seedlings were harvested after measuring photosynthetic rate and *F_v_*/*F_m_*. For seedling growth parameters, seedlings were divided into shoots and roots. Seedling length, seedling fresh weight, and root fresh weights were measured and recorded. Thereafter, the roots were scanned with an Epson Perfection V850 Pro root scanner (Regent Instruments Inc., Quebec City, QC, Canada) at a resolution of 600 dpi. Root morphological parameters including root length, surface area, root volume, average root diameter, root tips, root crossings, and root forks were measured by a WinRHIZO^™^ Pro image analysis system (Regent Instruments Inc., Quebec City, QC, Canada). The shoots and roots were then subsequently dried in a forced air oven (Sheldon Manufacturing Inc., Cornelius, OR, USA) at 65 °C for 72 h, and dry weight was measured. 

#### 2.2.3. Chlorophyll Contents

Leaf chlorophyll pigments (Chl *a* and Chl *b*) in growing seedling’s leaves were analyzed following the method developed by Minocha et al. [45]. Briefly, the fresh seedling leaves (100 mg) from each treatment were cut and submerged into 5 mL of 95% (*v*/*v*) ethanol in the dark and incubated at a temperature of 4 °C for 24 h. After that, the absorbance of extracted solution was recorded spectrophotometrically using a Cytation-5 microplate reader (BioTek Instruments, Santa Clara, CA, USA) at an absorbance of 649 nm and 665 nm, based on the equations used for Chl *a* and Chl *b* calculations. The chlorophyll contents were calculated according to the formulas of Lichtenthaler and Wellburn [46] and expressed as μg g^−1^ fresh weight (FW):

Chl a (μg/mL)=13.95×A665−6.88×A649

Chl b (μg/mL)=24.96×A649−7.32×A665 

### 2.3. Biochemical Analyses

#### 2.3.1. Proline Contents

Seedling leaf tissues (500 mg) were mixed with 5 mL of 3% (*w*/*v*) sulfosalicylic acid in 50 mL test tubes incubated at 100 °C in a water bath for 10 min [47]. A 2-mL aliquot of the cooled mixture was added to a mixture of 2 mL acetic acid and 3 mL ninhydrin solution, and the mixture heated at 100 °C for 40 min. After cooling, 4 mL toluene was added to each tube. The tube was then closed with a stopper and vortexed for 30 s followed by 60 min incubation in the dark. The proline–toluene phase (upper layer) was collected into a fresh tube, and the absorbance was measured at 520 nm using a Cytation−5 microplate reader. Proline contents were expressed as μg g^−1^ FW and calculated according to the proline standard curve (concentration between 0–20 μg/mL):

$$y=0.014\times x-0.029\;\;\;\;\;R^{2\;}=0.997$$

#### 2.3.2. Hydrogen Peroxide and Malondialdehyde Contents

Hydrogen peroxide (H_2_O_2_) content of the seedling leaf was determined as described by Othman et al. [48], with slight modifications. Briefly, seedling leaf samples (200 mg) were ground in 1.5 mL 0.1% trichloroacetic acid (TCA) solution, and the homogenate was centrifuged at 11,583 rpm for 20 min at 4 °C. Next, 0.5 mL supernatant was mixed with 0.5 mL 10 mM phosphate-buffered saline (PBS) solution (pH 7.0) and 1 mL 1M potassium iodide (KI) solution in a 2-mL centrifuge tube and then incubated at 28 °C. After 60 min of incubation, the absorbance at 390 nm wavelength was recorded in a microplate reader, and the H_2_O_2_ contents (μmol H_2_O_2_ g^−1^ FW) were calculated according to the following standard curve (concentration between 0–100 nmol/mL):

$$y=5.54\times x-0.004\;\;\;\;\;R^{2\;}=0.999$$

The level of seedling lipid peroxidation was determined by quantifying the malondialdehyde (MDA) content in leaf samples by adopting the method of Hodges et al. [49]. Briefly, 500 mg seedling leaf samples were homogenized in 5 mL of 10% (*w*/*v*) TCA and then centrifuged at 10,360 rpm for 20 min at 4 °C. Then, 2 mL supernatant was added into 2 mL 0.67% (*w*/*v*) thiobarbituric acid (TBA), then the mixture was kept in the boiling water for 15 min. The mixture was quickly chilled in the ice bath to stop the reaction. Afterward, the mixture was centrifuged at 11,583 rpm for 15 min. The absorbance was recorded at 440, 532, and 600 nm using a Cytation-5 microplate reader, and the MDA concentration (μmol L^−1^) was calculated adopting the following formula:

cMDA=6.452×(A532−A600)−0.56×A440

#### 2.3.3. Protein Extraction and Antioxidant Enzyme Assays

For crude protein extraction, a 200-mg seedling leaf sample was homogenized in a 1.5 mL extraction buffer, which contained 0.1 M PBS (pH 7.8), 2 mM ethylenediaminetetraacetic acid (EDTA), 2 mM ascorbic acid, and 1% (*w*/*v*) polyvinylpyrrolidone (PVP) using prechilled mortar and pestle. The homogenate was centrifuged at 12,000 rpm for 20 min at 4 °C, and the supernatant was collected for the protein assay and enzyme activity measurements, as explained below.

The protein content in seedling leaf samples was analyzed using a Cytation-5 microplate reader following the protocol of Bradford [50]. Briefly, 0.1 mL of crude protein supernatant was pipetted into a test tube, and 5 mL of Coomassie Brilliant Blue G-250 protein reagent were added. The contents were mixed well by shaking and then standing for 2–3 min. Protein concentration (mg g^−1^ FW) was determined at an absorbance of 595 nm based on the following standard curve (concentration between 0 and 100 μg/mL):

$$y=0.000528\times x-0.00456\;\;\;\;\;R^{2\;}=0.994$$

The method developed by Beauchamp and Fridovich [51] was followed to determine superoxide dismutase (SOD, EC. 1.15.1.1) activity in corn leaves. One unit of SOD activity was defined as causing 50% inhibition of nitro blue tetrazolium (NBT), measuring the absorbance of the chromophore at 560 nm. Aliquots (40 μL) of the enzyme extract were added to a 3 mL reaction mixture (50 mM PBS (pH 7.8), 2.25 mM NBT, 60 μM riboflavin, 3 mM EDTA, 14.5 mM methionine) and the reaction conducted in a light incubator under 4000 lux at 25 °C for 20 min. The unit of SOD activity was expressed in unit mg^−1^ protein.

The assay of catalase (CAT, EC. 1.11.1.6) activity was conducted following the method developed by Cakmak and Marschner [52]. Briefly, 0.1 mL of enzyme extract was added in the 2.9 mL reaction mixture containing 0.15 M PBS (pH 7.0) and 10 mM H_2_O_2_. The absorbance at 240 nm was recorded for 120 s in a UV-visible spectrophotometer (Agilent Technologies Inc., Santa Clara, CA, USA). The CAT activity was expressed for the decomposition of 1 μmol of H_2_O_2_ per minute (μmol mg^−1^ protein min^−1^). 

Ascorbate peroxidase (APX, EC. 1.11.1.11) activity was measured according to the method developed by Nakano and Asada [53]. The reaction mixture was prepared by adding PBS (50 mM, pH 7.0), EDTA (0.1 mM), ascorbic acid (AsA, 5 mM), and H_2_O_2_ (20 mM), and then, 0.1 mL of supernatant was added into a 2.9 mL reaction mixture. The absorbance at 290 nm was recorded for 120 s in a UV-visible spectrophotometer (Agilent Technologies Inc., Santa Clara, CA, USA). The APX activity was expressed in the amount of enzyme that can oxidize 1 μmol of AsA per minute (μmol mg^−1^ protein min^−1^). 

### 2.4. Statistical Analyses

The experimental design was as follows: There were three biological replicates for each experimental unit and each biological replicate had at least three technical replicates. The experiment was repeated twice independently. Both independent experiments expressed the same trends, therefore, the data were pooled and analyzed. The Shapiro–Wilk test was employed on the data to check the normality before further statistical analysis was conducted. The XLSTAT program (Permium 2020, Addinsoft Inc., New York, NY, USA) was used for two-way analysis of variance (ANOVA) with three sources of variation (genotypes, temperatures, and their interactions), and means were compared using Fisher’s Least Significant test (*p* ≤ 0.05). All data in this study were expressed as means ± standard errors (SEs, *n* = 6). Figures were prepared using GraphPad Prism (version 8.4.3, GraphPad Software, San Diego, CA, USA). Principal Component Analysis (PCA) and its visualization were performed using the XLSTAT program to determine the relationships between temperatures, genotypes, morphological, physiological, and biochemical attributes. R statistic software was used to conduct Pearson’s correlation analysis [54]. 

## 3. Results

### 3.1. Seedling Growth and Root Characteristics

Temperature regime significantly (*p* < 0.001) affected the seedling shoot length, shoot dry weight, root dry weight, and seedling dry weight of silage corn genotypes. However, the interaction was only significant in the case of shoot length (Table 1). Significantly higher shoot dry weight (0.95 ± 0.02 g seedling^−1^), root dry weight (0.25 ± 0.01 g seedling^−1^), and seedling dry weights (1.20 ± 0.03 g seedling^−1^) were recorded at 25 °C, whereas the lowest values were observed at 5 °C (Table 1). Overall, the evaluated growth parameters varied in the order of 25 °C > 20 °C > 15 °C > 10 °C > 5 °C (Table 1). The Yukon-R silage corn genotype produced seedlings with significantly higher shoot dry weight (0.75 ± 0.03 g seedling^−1^), root dry weight (0.22 ± 0.01 g seedling^−1^), and total dry weight (0.97 ± 0.03 g seedling^−1^) compared to genotype A4177G3-RIB. (Table 1). Interaction among growth temperature and silage corn genotypes displayed maximum shoot length (80.02 ± 1.30 cm seedling^−1^) in Yukon-R when grown at 25 °C, while the lowest shoot length (44.99 ± 0.52 cm seedling^−1^) was noted in A4177G3-RIB at 5 °C (Table 1). 

**Figure 1:**
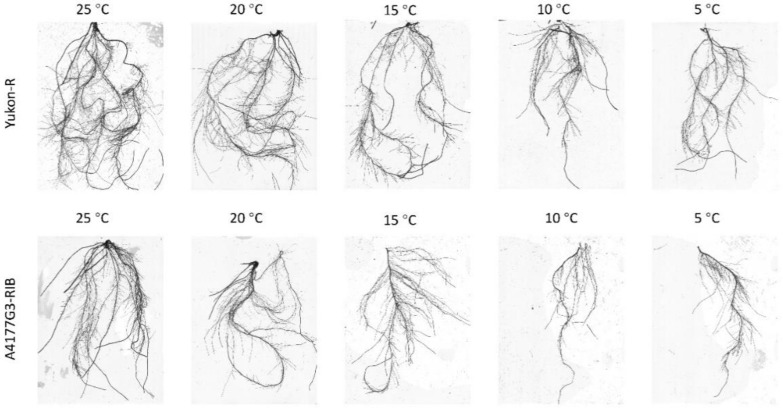
Effect of chilling temperature (5, 10, and 15 °C) and non-chilling temperature (20 and 25 °C) on silage corn seedling root growth.

Temperature regimes significantly (*p* < 0.05) effected the total root length, individual root length (0–0.5 mm and >0.5 mm), total root volume and individual root volume (0–0.5 mm and >0.5 mm), total root surface area and individual root surface area (0–0.5 mm and >0.5 mm), and total root forks and total root crossings of silage corn genotypes (Table 2 and Table 3). The interactions between growth temperature and silage genotype were significant in root length (>0.5 mm), root volume (0–0.5 mm, >0.5 mm), and root surface area (0–0.5 mm and >0.5 mm), as shown in Table 2. Silage corn seedlings grown at 25 °C had significantly longer roots (1416 ± 59 cm seedling^−1^), higher root volume (2.24 ± 0.05 cm^3^ seedling^−1^), total root surface area (158.19 ± 3.19 cm^2^ seedling^−1^), average root diameter (0.366 ± 0.007 mm seedling^−1^), total root tips (3649 ± 358 seedling^−1^), root tips at 0–0.5 mm (3592 ± 346 seedling^−1^), total root forks (5341 ± 211 seedling^−1^), and total root crossings (1298 ± 65 seedling^−1^), whereas the lowest values were noticed at 5 °C, irrespective of silage corn genotypes (Table 2 and Table 3, Figure 1). Yukon-R had significantly longer roots (1108 ± 69 cm seedling^−1^), higher root volume (1.48 ± 0.06 cm^3^ seedling^−1^), total root surface area (104.55 ± 6.19 cm^2^ seedling^−1^), higher root forks (4090 ± 290 seedling^−1^), and root crossings (1009 ± 79 seedling^−1^) than A4177G3-RIB (Table 2 and Table 3). Overall, all the root traits varied in order of the following temperature: 25 °C > 20 °C > 15 °C > 10 °C and > 5 °C, irrespective of silage corn genotypes. However, Yukon-R showed superior shoot growth and root morphological traits than A4177G3-RIB (Table 2 and Table 3, Figure 1). 

### 3.2. Chlorophyll Content, Photosynthesis Rate, and Maximum Photochemical Efficiency of PS-II

**Figure 2:**
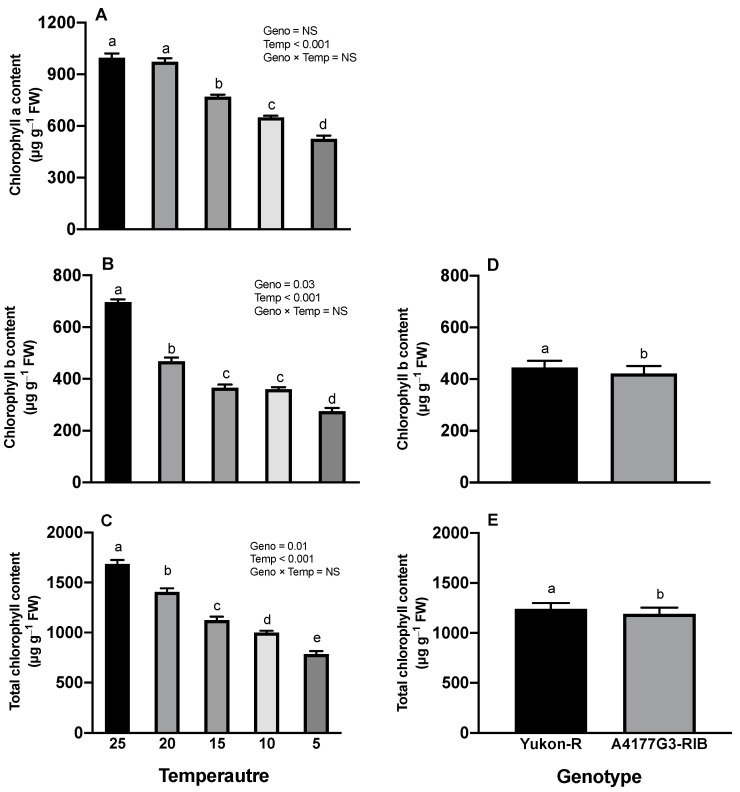
Effects of temperature regime on leaf chlorophyll (**A**), chlorophyll *b* (**B**), and total chlorophyll content (**C**) of silage corn genotypes; the response of silage corn genotypes to chlorophyll *b* content (**D**) and total chlorophyll content (**E**). Each vertical bar represents the average of replicates ± SE of the mean (*n* = 30 for Geno, *n* = 12 for Temp, *n* = 6 for Geno × Temp). Different letters indicate significant differences among treatments at *p* ≤ 0.05 according to Fisher’s Least Significant test. Geno = genotype; Temp = temperature.

Growth temperatures had significant effects (*p* < 0.001) on total chlorophyll content, and Chl *b* content on silage corn seedlings, whereas Chl *a* content is affected (*p* < 0.001) by growth temperatures only (Figure 2A–E). However, the interactions between growth temperatures and silage corn genotypes had no effects on Chl *a*, Chl *b*, and total chlorophyll contents (data not shown). Significantly higher total chlorophyll (1687.62 ± 32.83 μg g^−1^ FW), Chl *a* (996.58 ± 23.95 μg g^−1^ FW), and Chl *b* (696.32 ± 11.38 μg g^−1^ FW) were observed at 25 °C whereas the lowest values were recorded at 5 °C (Figure 2A–C). Yukon-R genotype had significantly higher total chlorophyll (1241.73 ± 57.38 μg g^−1^ FW) and Chl *b* (444.67 ± 26.33 μg g^−1^ FW) than genotype A1477G3-RIB (Figure 2D,E). 

**Figure 3:**
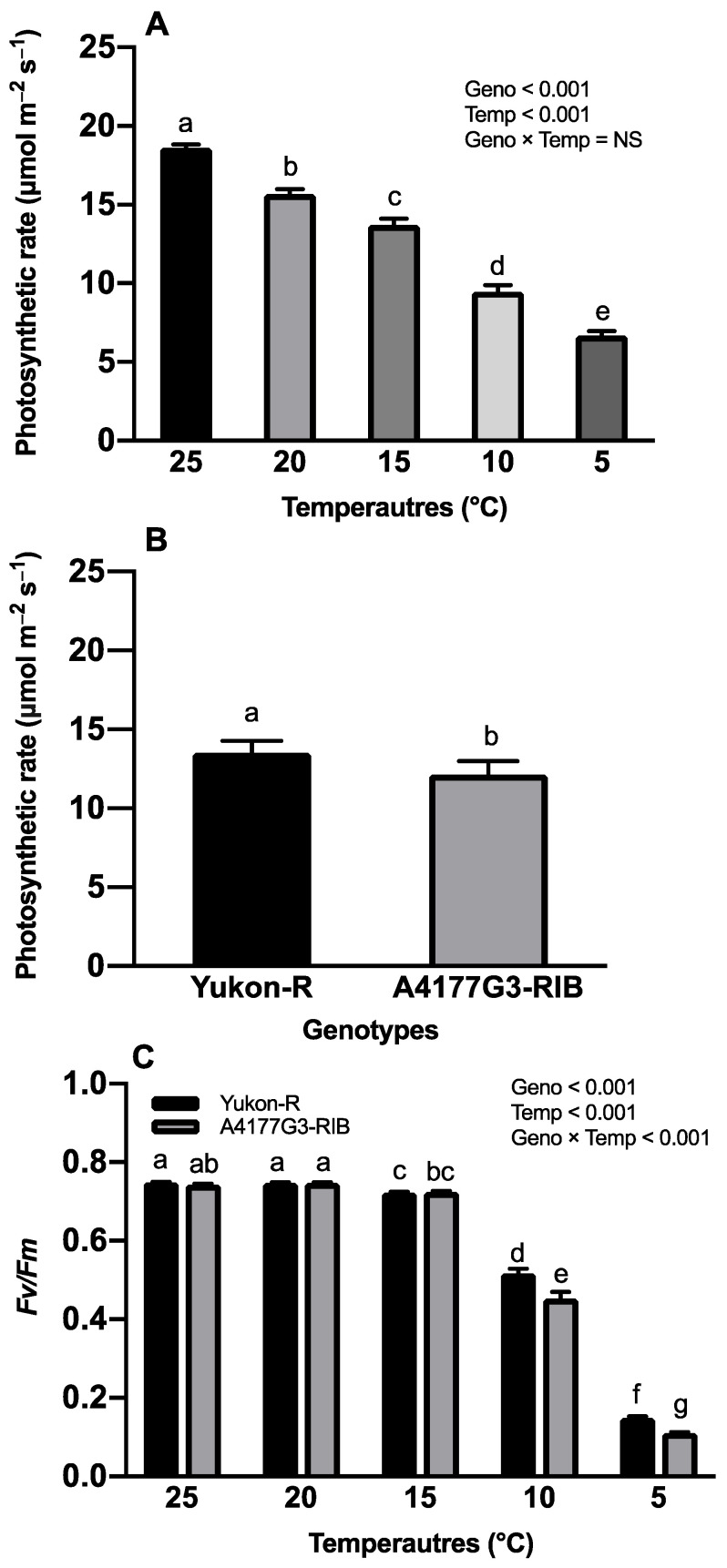
Effects of temperature regime on photosynthesis rate of silage corn (**A**). Comparative photosynthesis rate of silage corn genotypes (**B**)**.** Interactive effects of temperature and silage corn genotypes on photochemical efficiency of PS-II (*F_v_*/*F_m_*) (**C**). Each vertical bar represents the average of replicates ± SE of the mean (*n* = 30 for Geno, *n* = 12 for Temp, *n* = 6 for Geno × Temp). Different lowercase letters indicate significant differences among treatments at *p* ≤ 0.05 according to Fisher’s Least Significant test. Geno = genotype; Temp = temperature.

In this study, photosynthesis rate is a measure of the net CO_2_ assimilation rate of silage corn leaf, which is computed as the rate of CO_2_ uptake per unit time per unit leaf area (μmol m^−2^ s^−1^). Growth temperatures expressed significant (*p* < 0.001) effects on the photosynthesis rate of silage corn genotypes; however, the interaction (temperature regime × corn genotypes) had significant effects on the photosynthetic efficiency of PS-II (Figure 3C). A significantly higher photosynthesis rate (18.60± 0.23 μmol m^−2^ s^−1^) was observed at 25 °C whereas the lowest was recorded at 5 °C (6.68 ± 0.30 μmol m^−2^ s^−1^), irrespective of silage corn genotypes (Figure 3A). Yukon-R showed a significantly higher photosynthesis rate (13.51 ± 0.77 μmol m^−2^ s^−1^) than A4177G3-RIB (12.14 ± 0.85 μmol m^−2^ s^−1^) (Figure 3B). Temperature × corn genotype interaction resulted in significantly higher *F_v_*/*F_m_* values in Yukon-R (0.75 ± 0.01) at 25 °C. However, both genotypes had similar *F_v_*/*F_m_* values when exposed to 20 °C (Figure 3C). 

### 3.3. Hydrogen Peroxide and Malondialdehyde Contents 

**Figure 4:**
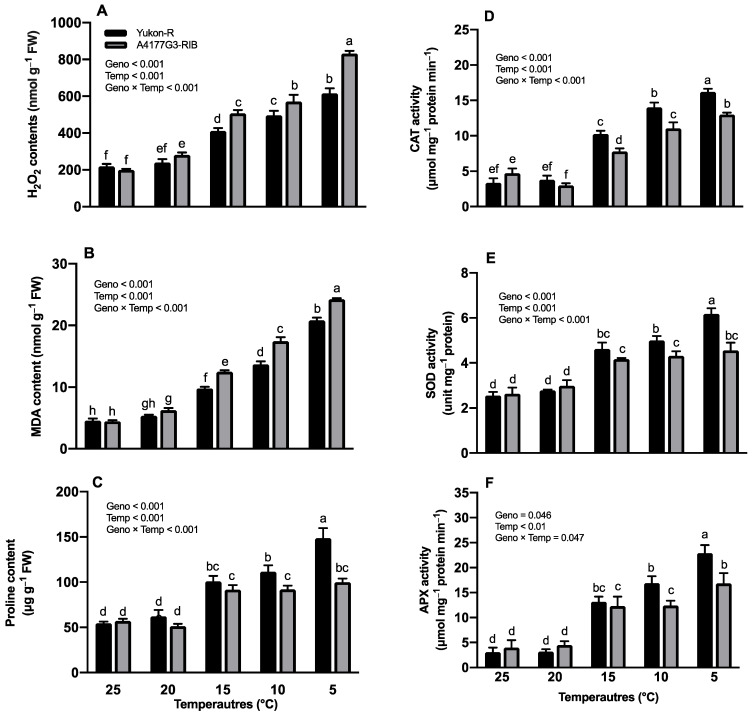
Interactive effects of temperature regime and silage corn genotypes on leaf H_2_O_2_ content (**A**), MDA contents (**B**), proline content (**C**), SOD activity (**D**), CAT activity (**E**), and APX activity (**F**). Each vertical bar represents the average of replicates ± SE of the mean (*n* = 6). Different lowercase letters indicate significant differences among treatments at *p* ≤ 0.05, according to Fisher’s Least Significant test. Geno = genotype; Temp = temperature. H_2_O_2_, MDA, SOD, CAT, and APX represent hydrogen peroxide content, malondialdehyde, superoxide dismutase, catalyze activity, and ascorbate peroxidase activity.

We found that growth temperature, silage corn genotype, and their interactions (Geno × Temp) had a significant difference (*p* < 0.001) in both H_2_O_2_ and MDA contents of silage corn seedlings. Significantly higher (831.80 ± 11.91 nmol g^−1^ FW) H_2_O_2_ accumulation was observed in A4177G3-RIB seedlings at 5 °C whereas the lowest (199.39 ± 5.93 nmol g^−1^ FW) was noticed in the same genotypes at 25 °C, as shown in Figure 4A. Similarly, the MDA content was significantly higher (24.23 ± 0.18 nmol g^−1^ FW) in A4177G3-RIB at 5 °C and the lowest (4.47 ± 0.18 nmol g^−1^ FW) was recorded in the same genotype at 25 °C (Figure 4B). Both genotypes produced statistically similar H_2_O_2_ and MDA contents at 25 and 20 °C conditions, however, the MDA contents increased dramatically at 15 °C. While comparing the main effects, genotypes expressed higher H_2_O_2_ and MDA contents in the order of 25 °C > 20 °C > 15 °C > 10 °C and > 5 °C whereas A4177G3-RIB expressed higher content than Yukon-R (data not shown).

### 3.4. Proline Contents and Antioxidant Enzyme Activities

Temperature regime, silage corn genotypes, and their interaction (Geno × Temp) had significant (*p* < 0.001) effects on proline contents (Figure 4C). Significantly higher proline content (148.58 ± 11.18 ug g^−1^ FW) was observed in Yukon-R when grown at 5 °C, whereas the lowest was recorded in A4177G3-RIB at 20 °C (51.49 ± 2.48 ug g^−1^ FW) (Figure 4C). Proline contents were observed in the order of 5 °C < 10 °C < 15 °C < 20 °C and 25 °C while comparing the effects of temperature regime, and Yukon-R expressed higher proline content compared to A4177G3-RIB (data not shown). 

Temperature regimes, silage corn genotypes, and their interaction (Geno × Temp) expressed significant (*p* < 0.001) effects on antioxidants (CAT, APX, and SOD) (Figure 4D–F). Interactive effects of temperature regime and silage corn genotypes displayed significantly higher CAT (16.23 ± 0.76 μmol mg^−1^ protein min^−1^), APX (22.89 ± 1.61 μmol mg^−1^ protein min^−1^), and SOD (6.18 ± 0.25 unit mg^−1^ protein) values in the Yukon-R genotype when grown at 5 °C (Figure 4D–F). The lowest CAT activity (3.0 ± 0.14 μmol mg^−1^ protein min^−1^) was found at 20 °C in A4177G3-RIB, whereas the Yukon-R had the lowest SOD (2.60 ± 0.07 unit mg^−1^ protein) and APX (3.06 ± 0.42 μmol mg^−1^ protein min^−1^) activities at 25 °C. While comparing the main effects, CAT, APX, and SOD varied in order of 5 °C > 10 °C > 15 °C > 20 °C > 25 °C, irrespective of silage corn genotypes (Figure 4D–F).

### 3.5. Relationships between Morphological, Physiological, and Biochemical Attributes of Silage Corn Genotypes as Influenced by Different Temperature Regiems

**Figure 5:**
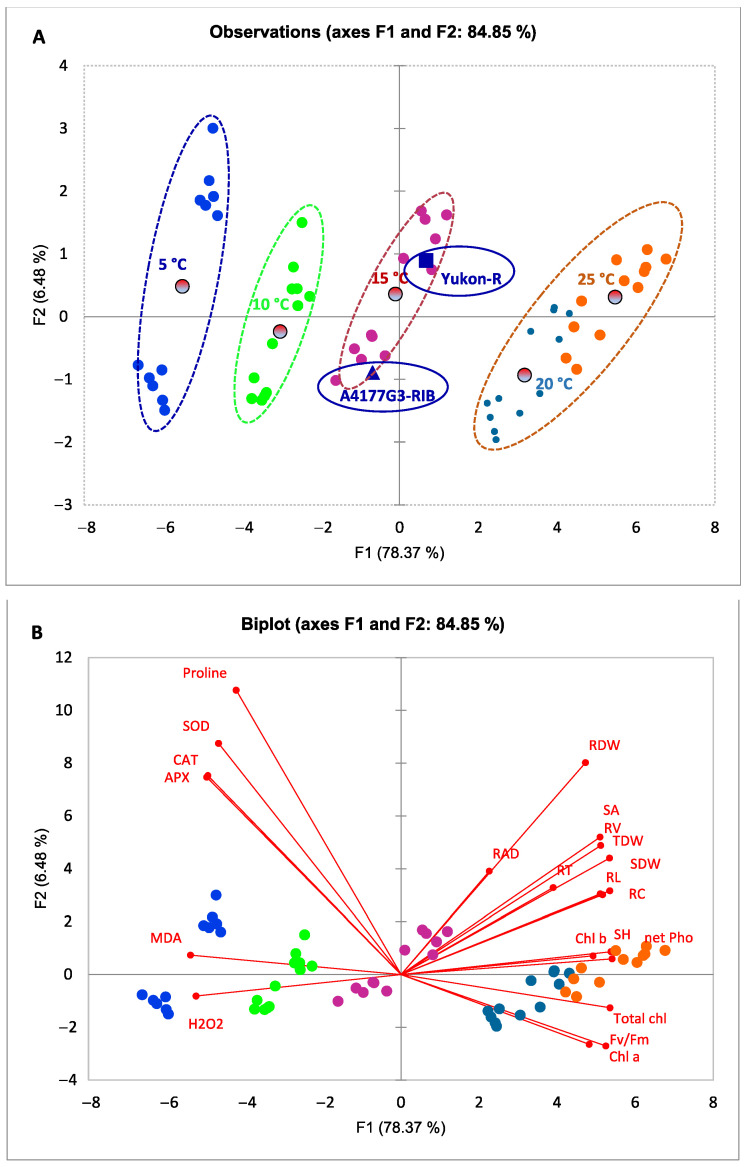
Observation plot showing separation of different temperature and silage corn genotypes in different quadrants (**A**). PCA biplot showing the association of morphological, physiological, and biochemical attributes of silage corn genotypes grown at different temperatures (**B**). APX: ascorbate peroxidase; CAT: catalase; SOD: superoxide dismutase; Chl *a*: chlorophyll *a*; Chl *b*: chlorophyll *b*; H_2_O_2_: hydrogen peroxide; MDA: malondialdehyde; RDW: root dry weight; RL: root length; RSA: root surface area; RV: root volume; SDW: shoot dry weight; SH: seedling height; total Chl: total chlorophyll.

PCA was performed to assess the association between experimental treatments and morphological, physiological, and biochemical attributes of corn genotypes. The PCA explained 84.85% variability in the total data, where the first dimension (F1) displayed 78.38% and the second dimension (F2) showed 6.48% variability, respectively (Figure 5A,B). The studied parameters grouped temperature regimes in different quadrants (Figure 5A,B). The 21 different parameters separated the temperature treatments and two silage corn genotypes into four quadrants (Q) of the PCA. The observation plot showed a clear segregation of five temperature regimes where 25 °C and 20 °C were grouped in the right region of the F1 (Q3 and Q4), whereas 10 °C and 5 °C were observed in the right region of the F1 (Q1 and Q3) and 15 °C was grouped in the intermediate region of these groups (Figure 5A). We also observed clear segregation of two silage corn genotypes: Yukon-R was shown in the Q3, whereas A4177G3-RIB in the Q2 (Figure 5A). The biplot showed that the silage corn’s morphological and photosynthetic parameters had a strong positive relationship. However, the biochemical attributes were shown in the opposite region, suggesting that low temperatures enhanced ROS accumulation and antioxidant activities, whereas they reduced seedling growth. All biochemical attributes were observed in the same quadrant, and ANOVA results showed an increasing trend due to temperature drop. Moreover, APX, CAT, SOD, and proline were strongly associated with Yukon-R under 5 °C and 10 °C treatment, indicating Yukon-R has better chilling tolerance capacity than A4177G3-RIB (Figure 5B). 

**Figure 6:**
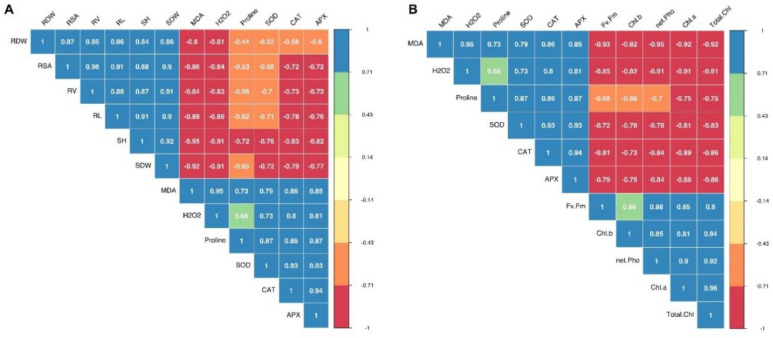
The Pearson’s correlation matrixes showing association between the different parameters in silage corn genotypes when grown at low temperatures. Correlation matrix between morphological and biochemical attributes of silage corn (**A**); correlation matrix between silage corn photosynthetic and biochemical parameters (**B**). APX: ascorbate peroxidase; CAT: catalase; SOD: superoxide dismutase; Chl *a*: chlorophyll *a*; Chl *b*: chlorophyll *b*; H_2_O_2_: hydrogen peroxide; MDA: malondialdehyde; RDW: root dry weight; RL: root length; RSA: root surface area; RV: root volume; SDW: shoot dry weight; SH: seedling height; total Chl: total chlorophyll.

Pearson’s correlation analysis showed negative correlations between silage corn morphological parameters, including RDW, RSA, RV, RL SH, SDW, MDA, H_2_O_2_, proline, CAT, and APX (Figure 6A). Similarly, the photosynthetic parameters (*F_v_*/*F_m_*, Chl *a*, Chl *b*, total chlorophyll, and photosynthetic rate) were all negatively correlated with the biochemical parameters (Figure 6B). For instance, the correlation shows that ROS accumulation (H_2_O_2_, MDA) and plant growth parameters (morphological and physiological) are strongly negatively correlated (Figure 6A,B). For the morphological parameters, the highest negative correlation (−0.95) was found between shoot height and MDA content (Figure 6A). For the photosynthetic parameters, the highest negative correlation (−0.95) was observed between photosynthetic rate and MDA content, as shown in Figure 6B. 

## 4. Discussion

### 4.1. Changes in Silage Corn Seedling Growth and Photosynthetic Capacity in Response to Different Temperatures

Low-temperature stress is one of the major abiotic factors which adversely affect seedling establishment and forage production in boreal agroecosystem [41,55,56]. In the present study, low/chilling temperature (15 °C, 10 °C, and 5 °C) significantly reduced seedlings’ growth, dry weight, and root morphological traits (Figure 1) (Table 1, Table 2 and Table 3), total chlorophyll contents, chlorophyll *a* and *b*, photosynthesis rate, and *F_v_*/*F_m_* (Figure 2A–C and Figure 3A,C) in both genotypes. This reduction in seedling and root morphological traits under low-temperature stress can be attributed to decreased cell division and elongation [57], photosynthetic capacity [23,58], and reduced water, nutrient uptake, and nutrient use efficiency [13,59]. 

Despite the fact that we did not analyze the nutrient uptake, the reduced seedling shoot biomass indicated lower seedling growth, which might be associated with lower nutrient and water uptake. Hund et al. [60] demonstrated that the root morphological traits play a key role in determining corn chilling tolerance capacity, especially the primary lateral root length. In the present study, we also observed a significant reduction in silage corn root parameters including root length, surface area, and volume in both cultivars in response to chilling stress. However, genotype Yukon-R maintained higher root morphological parameters than A4177G3-RIB by producing mainly fine roots (Figure 1, Table 2). These results suggested that a better root system of Yukon-R supports higher biomass production under chilling conditions (Figure 1, Table 1, Table 2 and Table 3). In addition, reduced root growth affects mineral and water absorption and transportation, which results in lower aboveground biomass [61,62]. In our study, we observed 38.7%, 48.4%, 40%, and 46.7% reduced shoot length, shoot dry weight, root dry weight, and total seedling dry weight, respectively, at 5 °C compared to 25 °C (Table 1). The higher biomass production of Yukon-R than A4177G3-RIB might be associated with its higher stress tolerance capacity to perform better under lower growth temperatures of 5 °C or 10 °C. 

The chlorophylls are involved in the initial event of photosynthesis, including light absorbing, energy transfer, and light energy transducing [63,64]. Many studies indicated the negative impacts of chilling stress on chlorophyll contents [12,65,66]. It is reported that reduction in chlorophyll content levels can increase the level of energy dissipation, which decreases PS-II efficiency [67,68]. However, reduced chlorophyll content is not involved in chloroplast development [69], which also might improve photosynthetic light use efficiency by increasing light penetration and distribution within the canopy [70]. The decrease in chlorophyll contents under chilling stress was mainly due to the chlorophyll biosynthesis disruption and chlorophyll degradation. Zhao et al. [71] reported lower activities of glutamate-1-semialdehyde transaminase, magnesium chelatase, and protochlorophyllide oxidoreductase when rice plants were grown at 12 °C for 48 h during the greening phase. In the present study, we also observed that chilling stress caused a significant reduction in Chl *a*, Chl *b*, and total chlorophyll content (Figure 2 and Figure 3). The Chl *a*, Chl *b*, and total chlorophyll contents were markedly reduced by 47.2%, 60.5%, 53.4%, respectively, when the seedlings were exposed to 5 °C compared to 25 °C (Figure 2 and Figure 3). Lidon et al. [72] also reported reduced chlorophyll pigments due to chilling stress. Therefore, the reduced Chl *a*, Chl *b*, and total chlorophyll content in our study might be associated with lower enzyme activities, as reported by Zhao et al. [71]. 

C_4_ plants such as corn are considered efficient in water and nitrogen use compared to C_3_ plants. However, their photosynthetic system is strongly affected by chilling temperatures [18,73]. Kubien et al. [74] indicated that C_4_ plants have less ribulose-1,5-bisphosphate carboxylase (Rubisco) content than C_3_ plants in cool climates. Therefore, Rubisco capacity is the key factor limiting the C_4_ photosynthesis rate under chilling conditions [44].

Under the chilling stress, a 60% reduction in the CO_2_ assimilation capacity has been reported in the growing plants, thus causing a lower growth rate and grain yield [18]. Compared to the 25 °C, low-temperature treatments of 15 °C, 10 °C, and 5 °C significantly reduced net photosynthesis rates by 26.4%, 49.1%, and 64.1%, respectively (Figure 3A). The decreased photosynthesis rate is attributed to chilling-induced stomal closure and loss of enzyme activities of CO_2_ concentration mechanisms [75,76]. Here, we also found that the photosynthesis rate significantly decreased when silage corn seedlings were exposed to lower than 20 °C for 5 days, which showed the high sensitivity of silage corn in response to temperature decrease. Yukon-R had a 10% higher photosynthesis rate than A4177G3-RIB (Figure 3B), which suggested that Yukon-R has a greater potential for forage crop production in cool climates due to its high CO_2_ fixation rate. 

The *F_v_*/*F_m_* ratio represents the maximal photochemical efficiency of PS-II, which is widely used in detecting the PS-II photoinhibition induced by chilling stress in many plant species [76,77,78,79]. Chilling stress causes a decline of this ratio mainly from decreasing light absorption in the thylakoid electron transport of PS-II and increasing excitation energy quenching in the light-harvesting antennae [21,80]. Moreover, chilling-induced ROS (H_2_O_2_ and O_2_^•−^) can damage PS-II, and the biosynthesis of D1 protein for PS-II repair during chilling conditions is also inhibited [61,81]. Chlorophyll fluorescence analysis is considered a valid tool for selecting chilling tolerant corn genotypes [60,82]. Recently, Yi et al. [83] reported that most quantitative trait loci (QTLs) for cold tolerance were associated with the ratio of *F_v_*/*F_m_* in corn. In the present study, we found that low-temperature/chilling stress of 5 °C and 10 °C significantly reduced the ratio of *F_v_*/*F_m_* in both genotypes (Figure 3A); similar results were also reported from previous studies [84,85]. Moreover, the interactions between temperature and genotype were significant. The *F_v_*/*F_m_* ratio in Yukon-R was significantly higher than A4177G3-RIB under severe chilling stress conditions of 5 °C and 10 °C (Figure 3C), which could be attributed to the higher efficiency of energy transfer and less damage of PS-II in Yukon-R under chilling stress [17]. Interestingly, at a mild chilling temperature (15 °C) condition, there was no significant difference of *F_v_*/*F_m_* values between the two genotypes, and the decline of *F_v_*/*F_m_* was slightly less compared with the control (Figure 3C). This might be due to silage corn’s acclimation to the mild chilling stress conditions [86]. 

### 4.2. Chilling Stress-Induced Osmotic Stress, ROS, and Activity of Antioxidant Enzymes in Silage Corn Seedlings

The measurement of MDA content has been used as a lipid peroxidation biomarker in many studies related to cold stress response [87,88,89]. The over-accumulation of ROS during chilling stress can cause lipid peroxidation, which increases membrane leakage and decreases membrane fluidity, which is also considered the main reason for damaged membrane-localized proteins associated with ion-channels, receptors, and enzyme architecture [27]. Polyunsaturated fatty acids, such as linoleic and linolenic acid, are prone to attack by ROS under chilling stress. In addition, MDA strongly affects biomolecules and stress-related gene expression in plant cells [90,91]. Erdal [92] reported that the MDA and H_2_O_2_ contents increased by 27.5% and 22.6%, respectively, under chilling temperatures (10 °C/7 °C day/night) for 3 days. Huang and Guo [93] indicated that the oxidative stress levels are different between rice cultivars with different chilling sensitivity, and the lower ROS and MDA contents were highly associated with chilling stress tolerance. In the present study, low/chilling temperature stress treatments increased H_2_O_2_ and MDA contents in silage corn seedlings; the interactive effects of temperature and genotype on H_2_O_2_ and MDA contents were significant (Figure 4A,B). We have also found that the H_2_O_2_ and MDA contents of A4177G3-RIB were 35.2% and 16.5%, respectively, higher than Yukon-R at 5 °C. The generation of H_2_O_2_ occurs in various cellular sites in the plant cell, including the chloroplast, peroxisome, mitochondria, plasma membrane, and in the cytoplasm [94]. Furthermore, chilling stress limits CO_2_ fixation and NADP^+^ generation, which induces over-reduction of the photosynthetic electron transport chain [81,95]. It results in the accumulation of O_2_^•−^ in the chloroplast, and H_2_O_2_ is accumulated by the action of SOD [96]. Chilling stress also can affect the equilibrium in the production and scavenging of H_2_O_2_, and activities of scavenging enzymes such as APX maybe suppressed [97]. Thus, the present study suggests that Yukon-R has a higher chilling stress tolerance capacity due to its lower ROS and MDA accumulations than A4177G3-RIB. 

The osmotic potential could be severely affected when plants are exposed to chilling stress conditions, and wilting is one of the visible symptoms caused by chilling temperatures [98]. Proline is an important osmoprotectant and signaling molecule to avoid chilling injury induced by chilling stress [12,99]. Proline can prevent protein aggregation during protein refolding, which shows the critical role of proline in stabilizing and enhancing antioxidant enzyme activities under stress conditions [100,101]. Previous studies also revealed the function of proline in scavenging ROS, such as ^1^O_2_ [102,103,104]. Moreover, several studies suggested that exogenous application of proline or modification of its responsive genes are effective strategies to improve chilling tolerance [105]. In the present study, the interaction between temperature and genotype significantly affects proline content in silage corn leaf tissues. Compared to the non-chilling temperature conditions (20 °C and 25 °C), proline content increased dramatically during chilling stress in silage corn seedlings (Figure 4C). Proline contents in Yukon-R at 15 °C, 10 °C, and 5 °C treatments were significantly increased by 84.5%, 104.1%, and 172.1%, respectively (Figure 4C). The main role of increased accumulation of free proline is probably attributed to increasing osmotic potential to avoid chilling-induced dehydration in silage corn seedlings [106]. In addition, proline may contribute to scavenging of ROS, stabilizing membrane and proteins, as well as regulating the proteins synthesis under chilling stress [105,107]. The result also suggests that Yukon-R has superior osmotic regulatory ability in response to chilling response.

Chilling stress contributes to the over-accumulation of ROS in growing plants [92,97]. The increased enzymatic antioxidant activities are vital to minimize the ROS toxicity in plant cells under chilling stress conditions. Moreover, transcript levels of antioxidant enzymes have also been found to be up-regulated by low temperatures, and transgenic overexpression of antioxidant genes can also enhance plant chilling tolerance [27,108,109,110]. The PCA results also indicated positive effects of the chilling temperature regime group (10 °C and 5 °C) on antioxidant enzymes (Figure 5A,B). It is vital to balance the steady-state level between O_2_^•−^ and H_2_O_2_ in plant cells, which could prevent the generation of highly toxic hydroxyl free radicals by Harber–Weiss reaction and Fenton reaction under chilling stress conditions [111,112,113]. The SOD is ubiquitous in plant cells and acts as the first line in detoxifying ROS, which could catalyze O_2_^•−^ to H_2_O_2_ [25]. There are three isozymes of SODs based on their metal cofactors, which include Cu/Zn-SOD (cytosol, peroxisome, and chloroplast), Fe-SOD (chloroplast), and Mn-SOD (mitochondria) [27]. Once H_2_O_2_ was generated, the plants then required CAT and APX to metabolize it. The CAT plays an important role in decomposing H_2_O_2_ to H_2_O in the peroxisomes, which is highly expressed and has a fast reaction rate in plants [27,114]. The APX is involved in the water–water cycle and ascorbate–glutathione cycle in the chloroplast (sAPX) and cytosolic (cAPX), which utilizes AsA to reduce H_2_O_2_ to H_2_O [115]. Compared with CAT, APX is considered an effective H_2_O_2_ scavenger due to its better affinity for H_2_O_2_ [116]. The antioxidant defense system activation was observed and accompanied by chilling tolerance in many plant species, such as rice [76], corn [117,118], and wheat [119]. In the present study, our results indicated that the activities of CAT, SOD, and APX were all up-regulated under low/chilling temperature stress, and the interaction of temperature and genotype was significant (Figure 4D–F). Compared with A4177G3-RIB, Yukon-R had higher enzymatic activities under severe chilling stress, which is consistent with the lower H_2_O_2_ and MDA content we found in this genotype (Figure 4A–F). Moreover, the higher photosynthesis rate in Yukon-R may be attributed to higher SOD and APX activities in the chloroplast under chilling stress [81,120]. The up-regulation activity of CAT, SOD, and APX is considered an important strategy to alleviate chilling-induced oxidative stress, and some ROS molecules such as H_2_O_2_ also induce antioxidant enzyme related genes’ expression [121,122,123,124]. The balance of SOD, CAT, and APX is crucial for controlling the H_2_O_2_ level in a cell [115]. These antioxidant enzymes were positively correlated to corn chilling stress tolerance and were reported to be helpful in screening for chilling tolerance in different corn varieties [117,125,126].

## 5. Conclusions

In summary, the results from this study showed that low/chilling temperature stress induces significant morphological, physiological, and biochemical changes in silage corn seedlings at the early growth stage. Root growth and distribution, such as root weight, length, surface area, and root volume, were greatly reduced under stressed conditions, which ultimately inhibited silage corn aboveground growth. Moreover, chilling temperatures, particularly 10 °C and 5 °C, triggered higher ROS accumulation and lower photosynthetic capacity than non-chilling conditions. Silage corn genotypes exhibited differential tolerance capacity in response to chilling and non-chilling stress. Genotype Yukon-R produced seedlings with higher shoot growth/biomass and lower H_2_O_2_ and MDA contents than A4177G3-RIB under chilling stress. The higher photosynthesis, proline content, and antioxidant enzymatic activities contributed to improved seedling biomass and chilling tolerance in Yukon-R. Moreover, over-accumulation of ROS and lipid peroxidation at the early growth stage contributed to significant reductions in the growth and root morphological traits of silage corn seedlings under chilling stress condition. This study provides useful information on potential silage corn genotypes with cold tolerant traits that may be suitable for cultivation in boreal climates. Further research is needed to find the key regulators involved in the silage corn chilling stress response using appropriate approaches such as lipidomics and transcriptomics analyses.

## Figures and Tables

**Table 1:** Effects of temperature regime on seedling shoot length, shoot dry weight, root dry weight, and total seedling dry weight of two silage corn genotypes cultivated in hydroponics under controlled environmental conditions.

Source of Variance	Seedling Shoot Length (cm Seedling^−1^)	Shoot Dry Weight (g Seedling^−1^)	Root Dry Weight (g Seedling^−1^)	Seedling Ddry Weight (g Seedling^−1^)
**Temperature (Temp)**				
25 °C (control)	**77.41 ± 1.14 a**	**0.95 ± 0.02 a**	**0.25 ± 0.01 a**	**1.20 ± 0.03 a**
20 °C	72.66 ± 1.00 b	0.79 ± 0.01 b	0.22 ± 0.01 b	1.02 ± 0.02 b
15 °C	63.93 ± 1.84 c	0.70 ± 0.02 c	0.19 ± 0.01 c	0.89 ± 0.03 c
10 °C	53.79 ± 1.04 d	0.60 ± 0.02 d	0.16 ± 0.01 d	0.75 ± 0.03 d
5 °C	47.42 ± 0.95 e	0.49 ± 0.02 e	0.15 ± 0.01 e	0.64 ± 0.03 e
**Genotypes (Geno)**				
Yukon-R	**65.71 ± 2.11 a**	**0.75 ± 0.03 a**	**0.22 ± 0.01 a**	**0.97 ± 0.03 a**
A4177G3-RIB	60.37 ± 2.21 b	0.66 ± 0.03 b	0.16 ± 0.01 b	0.83 ± 0.04 b
**Temp × Geno**				
25 °C × Yukon-R	**80.02 ± 1.30 a**	0.98 ± 0.03	0.27 ± 0.01	1.25 ± 0.02
20 °C ×Yukon-R	73.25 ± 1.14 b	0.82 ± 0.03	0.25 ± 0.01	1.07 ± 0.03
15 °C × Yukon-R	68.74 ± 1.73 c	0.74 ± 0.02	0.23 ± 0.01	0.97 ± 0.02
10 °C × Yukon-R	56.71 ± 1.12 d	0.65 ± 0.02	0.19 ± 0.01	0.83 ± 0.02
5 °C × Yukon-R	49.87 ± 1.34 e	0.56 ± 0.02	0.18 ± 0.01	0.74 ± 0.02
25 °C × A4177G3-RIB	74.80 ± 1.96 b	0.91 ± 0.03	0.23 ± 0.01	1.14 ± 0.03
20 °C × A4177G3-RIB	72.07 ± 0.99 bc	0.78 ± 0.01	0.19 ± 0.01	0.96 ± 0.01
15 °C × A4177G3-RIB	59.12 ± 0.58 d	0.67 ± 0.02	0.16 ± 0.003	0.82 ± 0.02
10 °C × A4177G3-RIB	50.87 ± 1.17 e	0.54 ± 0.01	0.13 ± 0.004	0.68 ± 0.01
5 °C × A4177G3-RIB	44.99 ± 0.52 f	0.43 ± 0.01	0.12 ± 0.003	0.54 ± 0.01
**Significance**				
Temperatures	***	***	***	***
Genotypes	***	**	***	***
Temp × Gen	*	NS	NS	NS

*, **, *** represent significant differences at alpha 0.05, 0.01, and 0.001, respectively. NS represents not significant. The values presented here are means ± SE (*n* = 30 for Geno, *n* = 12 for Temp, *n* = 6 for Geno × Temp). Different letters within each column indicate significant differences among five growth temperatures, two silage corn genotypes, or their interaction according to Fishers’ Least Significant test (two-way ANOVA, *p* = 0.05).

**Table 2:** Analysis of variance and mean comparisons for total root length, total root volume, and total surface area of silage corn genotypes, grown in different temperatures and with their interaction under controlled environmental conditions.

Source of Variance	Total Root Length (RL) (cm Seedling ^−1^)	Root Length 0–0.5 mm (cm Seedling ^−1^)	Root Length >0.5 mm (cm Seedling ^−1^)	Total Root Volume (RV) (cm^3^ Seedling ^−1^)	Root Volume 0–0.5 mm (cm^3^ Seedling ^−1^)	Root Volume >0.5 mm (cm^3^ Seedling ^−1^)	Total Surface Area (SA) (cm^2^ Seedling ^−1^)	Surface Area 0–0.5 mm (cm^2^ Seedling ^−1^)	Surface Area >0.5 mm (cm^2^ Seedling ^−1^)
**Temperature (Temp)**									
25 °C (control)	**1416 ± 59 a**	**1145 ± 59 a**	**271 ± 8 a**	**2.24 ± 0.05 a**	**0.42 ± 0.02 a**	**1.82 ± 0.03 a**	**158.19 ± 3.88 a**	**64.21 ± 3.19 a**	**77.23 ± 2.54 a**
20 °C	1291 ± 49 a	1082 ± 47 a	209 ± 7 a	1.50 ± 0.07 b	0.36 ± 0.02 b	1.14 ± 0.08 b	121.31 ± 6.82 b	59.89 ± 3.81 a	47.79 ± 3.49 b
15 °C	966 ± 58 b	775 ± 50 b	189 ± 8 c	1.30 ± 0.05 b	0.33 ± 0.03 b	0.97 ± 0.05 c	109.33 ± 5.64 c	51.07 ± 3.88 b	46.28 ± 2.05 b
10 °C	612 ± 48 c	484 ± 45 c	127 ± 5 d	0.80 ± 0.03 c	0.19 ± 0.01 c	0.61 ± 0.03 d	66.68 ± 3.06 d	29.19 ± 2.09 c	30.13 ± 1.38 c
5 °C	589 ± 48 c	472 ± 35 c	116 ± 13 d	0.75 ± 0.04 c	0.17 ± 0.01 c	0.58 ± 0.06 d	61.45 ± 4.52 d	27.25 ± 1.68 c	27.85 ± 3.11 c
**Genotypes (Geno)**									
Yukon-R	**1108 ± 69 a**	**906 ± 60 a**	**201 ± 9 a**	**1.48 ± 0.06 a**	**0.34 ± 0.02 a**	**1.14 ± 0.08 a**	**104.55 ± 6.19 a**	**54.22 ± 3.56 a**	**50.33 ± 2.95 a**
A4177G3-RIB	842 ± 64 b	677 ± 52 b	164 ± 12 b	1.15 ± 0.06 b	0.24 ± 0.02 b	0.91 ± 0.09 b	79.80 ± 6.41 b	38.42 ± 2.61 b	41.38 ± 4.08 b
**Temp × Geno**									
25 °C × Yukon-R	1594 ± 41	1302 ± 41	**272 ± 15 a**	2.33 ± 0.08	**0.47 ± 0.02 a**	**1.86 ± 0.03 a**	148.04 ± 3.54	**72.56 ± 3.65 a**	75.47 ± 2.43 a
20 °C ×Yukon-R	1513 ± 59	1182 ± 67	223 ± 9 b	1.73 ± 0.08	0.43 ± 0.02 ab	1.31 ± 0.11 b	125.04 ± 5.56	70.52 ± 2.99 a	54.52 ± 3.66 b
15 °C × Yukon-R	1047 ± 66	904 ± 54	213 ± 7 bc	1.45 ± 0.06	0.41 ± 0.02 b	1.09 ± 0.05 c	113.62 ± 4.95	62.07 ± 3.03 b	51.56 ± 2.09 b
10 °C × Yukon-R	848 ± 33	581 ± 68	140 ± 9 e	0.88 ± 0.08	0.22 ± 0.02 ef	0.66 ± 0.02 fg	67.33 ± 3.19	34.56 ± 2.56 de	32.77 ± 1.26 de
5 °C × Yukon-R	608 ± 60	562 ± 47	158 ± 8 de	0.95 ± 0.04	0.19 ± 0.01 fg	0.77 ± 0.03 ef	68.73 ± 3.54	31.38 ± 2.24 e	37.35 ± 1.50 cd
25 °C × A4177G3-RIB	1284 ± 42	988 ± 65	269 ± 6 a	2.41 ± 0.03	0.36 ± 0.02 c	1.79 ± 0.06 a	134.85 ± 6.02	55.86 ± 1.89 bc	**78.99 ± 4.60 a**
20 °C × A4177G3-RIB	1186 ± 69	981 ± 38	195 ± 6 c	1.27 ± 0.04	0.30 ± 0.02 d	0.98 ± 0.09 cd	90.306 ± 7.24	49.25 ± 3.11 c	41.05 ± 4.71 c
15 °C × A4177G3-RIB	765 ± 25	647 ± 42	166 ± 7 d	1.11 ± 0.03	0.26 ± 0.02 de	0.85 ± 0.04 de	81.09 ± 3.16	40.07 ± 2.95 d	41.01 ± 1.72 c
10 °C × A4177G3-RIB	476 ± 54	387 ± 22	115 ± 6 f	0.71 ± 0.02	0.15 ± 0.01 g	0.55 ± 0.04 g	51.29 ± 2.31	23.81 ± 1.06 f	27.48 ± 2.00 e
5 °C × A4177G3-RIB	429 ± 18	343 ± 14	74 ± 7 g	0.54 ± 0.02	0.14 ± 0.0 g	0.39 ± 0.05 h	41.47 ± 1.81	23.11 ± 0.79 f	18.36 ± 2.09 f
**Significance**									
Temperatures	***	***	***	***	***	***	***	***	***
Genotypes	***	***	***	***	***	***	***	***	***
Temp × Gen	NS	NS	***	NS	***	*	NS	*	***

*, *** represents significant differences at alpha 0.05, 0.001, respectively. NS represents not significant. The values present here are means ± SE (*n* = 30 for Geno, *n* = 12 for Temp, *n* = 6 for Geno × Temp). Different letters within each column indicate significant differences among five growth temperatures, two silage corn genotypes, or their interaction according to Fishers’ Least Significant test (two-way ANOVA, *p* = 0.05).

**Table 3:** Analysis of variance and mean comparisons for root average diameters, total root tips, root forks, and root crossings of silage corn genotypes grown at different temperatures under a controlled environment.

Source of Variance	Root Average Diameters (mm)	Total Root Tips	Root Tips 0–0.05 mm	Root Tips >0.05 mm	Root Forks	Root Crossings
**Temperature (Temp)**						
25 °C (control)	**0.366 ± 0.007 a**	**3649 ± 358 a**	**3592 ± 346 a**	**39 ± 4 a**	**5341 ± 211 a**	**1298 ± 65 a**
20 °C	0.352 ± 0.004 a	2705 ± 144 b	2685 ± 144 b	19 ± 2 b	4702 ± 290 b	1233 ± 69 a
15 °C	0.350 ± 0.005 ab	2569 ± 222 b	2566 ± 220 b	30 ± 2 c	3908 ± 242 c	922 ± 65 b
10 °C	0.350 ± 0.009 ab	1794 ± 168 c	1778 ± 167 c	16 ± 3 c	2106 ± 166 d	427 ± 41 c
5 °C	0.332 ± 0.006 b	1684 ± 127 c	1676 ± 126 c	18 ± 2 c	1933 ± 124 d	411 ± 43 c
**Genotypes (Geno)**						
Yukon-R	0.35 ± 0.004	2665 ± 227	2631 ± 222	27 ± 2	**4090 ± 290 a**	**1009 ± 79 a**
A4177G3-RIB	0.34 ± 0.004	2310 ± 145	2288 ± 143	22 ± 2	3107 ± 258 b	707 ± 69 b
**Temp × Geno**						
25 °C × Yukon-R	0.376 ± 0.004	4248 ± 495	4174 ± 472	37 ± 6	5711 ± 238	1461 ± 58
20 °C ×Yukon-R	0.357 ± 0.004	2677 ± 276	2611 ± 274	16 ± 2	5351 ± 331	1391 ± 86
15 °C × Yukon-R	0.347 ± 0.005	2915 ± 349	2881 ± 345	34 ± 4	4632 ± 138	1119 ± 38
10 °C × Yukon-R	0.342 ± 0.016	1652 ± 267	1630 ± 264	21 ± 5	2508 ± 211	539 ± 41
5 °C × Yukon-R	0.343 ± 0.009	1833 ± 175	1809 ± 173	23 ± 2	2245 ± 167	534 ± 48
25 °C × A4177G3-RIB	0.356 ± 0.014	3050 ± 419	3009 ± 412	40 ± 7	4971 ± 291	1134 ± 68
20 °C × A4177G3-RIB	0.343 ± 0.005	2732 ± 125	2710 ± 125	21 ± 3	4054 ± 305	1075 ± 60
15 °C × A4177G3-RIB	0.356 ± 0.008	2277 ± 234	2251 ± 233	26 ± 2	3184 ± 174	724 ± 41
10 °C × A4177G3-RIB	0.357 ± 0.008	1937 ± 212	1926 ± 211	10 ± 1	1703 ± 112	314 ± 27
5 °C × A4177G3-RIB	0.321 ± 0.005	1556 ± 181	1542 ± 180	13 ± 2	1621 ± 33	288 ± 4
**Significance**						
Temperatures	*	***	***	***	***	***
Genotypes	NS	NS	NS	NS	***	***
Temp × Gen	NS	NS	NS	NS	NS	NS

*, *** represents significant differences at alpha 0.05 and 0.001, respectively. NS represents not significant. The values presented here are means ± SE (*n* = 30 for Geno, *n* = 12 for Temp, *n* = 6 for Geno × Temp). Different letters within each column indicate significant differences among five growth temperatures, two silage corn genotypes, or their interaction according to Fishers’ Least Significant test (two-way ANOVA, *p* = 0.05).

## Data Availability

The data that support the findings of this study are available from the corresponding author upon reasonable request.
